# Comparative RNA-Seq of Ten *Phaeodactylum tricornutum* Accessions: Unravelling Criteria for Robust Strain Selection from a Bioproduction Point of View

**DOI:** 10.3390/md22080353

**Published:** 2024-07-30

**Authors:** Charlotte Toustou, Isabelle Boulogne, Anne-Alicia Gonzalez, Muriel Bardor

**Affiliations:** 1 Laboratoire GlycoMEV UR 4358, Université de Rouen Normandie, SFR Normandie Végétal FED 4277, Innovation Chimie Carnot, 76000 Rouen, France; charlotte.toustou1@univ-rouen.fr; 2 MGX-Montpellier GenomiX, Univ. Montpellier, CNRS, INSERM, 34094 Montpellier, France; anne-alicia.gonzalez@inserm.fr; 3 ALGA BIOLOGICS, CURIB, 25 rue Tesnières, 76821 Mont-Saint-Aignan, France

**Keywords:** *P. tricornutum*, diatom, microalgae, RNA-Seq, bioproduction, biologics, pharmaceutical, recombinant proteins

## Abstract

The production of biologics in mammalian cells is hindered by some limitations including high production costs, prompting the exploration of other alternative expression systems that are cheaper and sustainable like microalgae. Successful productions of biologics such as monoclonal antibodies have already been demonstrated in the diatom *Phaeodactylum tricornutum*; however, limited production yields still remain compared to mammalian cells. Therefore, efforts are needed to make this microalga more competitive as a cell biofactory. Among the seventeen reported accessions of *P. tricornutum*, ten have been mainly studied so far. Among them, some have already been used to produce high-value-added molecules such as biologics. The use of “omics” is increasingly being described as useful for the improvement of both upstream and downstream steps in bioprocesses using mammalian cells. Therefore, in this context, we performed an RNA-Seq analysis of the ten most used *P. tricornutum* accessions (Pt1 to Pt10) and deciphered the differential gene expression in pathways that could affect bioproduction of biologics in *P. tricornutum*. Our results highlighted the benefits of certain accessions such as Pt9 or Pt4 for the production of biologics. Indeed, these accessions seem to be more advantageous. Moreover, these results contribute to a better understanding of the molecular and cellular biology of *P. tricornutum*.

## 1. Introduction

Biologics include a wide variety of drugs used to treat life-threatening diseases, ranging from gene or cell therapies to recombinant proteins. Today, recombinant protein therapies represent more than 60% of biopharmaceutical products and their peculiarity is that they must be produced by living systems, either mammalian cells or microorganisms, due to the complexity of the molecules that includes post-translational modifications [1,2,3]. Currently, recombinant proteins are mainly produced in mammalian systems, especially in CHO (Chinese Hamster Ovary) cells. Although considered as a gold standard, this type of production presents several drawbacks. The heterogeneity of the proteins and the possible contamination of the culture by viruses potentially transmissible to humans or by host cell proteins (HCPs) and DNA imply costly downstream processing steps in order to make the product compliant and safe according to pharmaceutical industry standards [4,5,6]. In this context, alternatives such as plants and microalgae are being explored [7,8,9,10]. Among these systems, microalgae seem to be advantageous due to their cheap culture conditions, their rapid growth rate in large-scale photobioreactors, and their ability to perform protein post-translation modifications [3,11,12,13,14]. In addition, using microalgae as cell biofactories to produce biologics contributes to the decarbonization of the pharmaceutical industry, especially biologics production. Moreover, some microalgae species, such as the diatom *Phaeodactylum tricornutum*, are considered safe by the Food and Drug Administration authority due to the lack of common pathogens between them and humans. Some of them have also been described as promising for the production of different biologics [3,7,9,12,15,16,17]. However, the use of microalgae for industrial-scale production of biologics is still hindered by their limited production yields, which currently do not reach the level of CHO cells. Therefore, much work needs to be performed to better understand the biology of these species in order to increase their biologics production yields.

The diatom *Phaeodactylum tricornutum* (Bohlin, 1897) has been described as a promising alternative for the production of biologics such as monoclonal antibodies (mAbs) directed against the antigens of deadly viruses [16,17,18] or recombinant antigens that have been used as sensitive diagnostic tools for the detection of the SARS-CoV-2 [19]. It is a well-established model diatom whose first genome sequencing and assembly in 2008 [20] allowed the development of powerful genetic tools like genome editing with TALENs [21] or CRISPR technologies [22,23,24]. In 2020, the sequencing of the ten most studied accessions of *P. tricornutum* (Pt1 to Pt10) revealed genetic diversity among these accessions, including CNVs (copy number variations) and INDELS (insertions and deletions) [25]. The analysis of molecular marker genes of these ten accessions allows their classification into four genetic clusters. In addition to genetic diversity, these accessions also differ phenotypically. Indeed, *P. tricornutum* has the unique ability to be pleiomorphic, and a single accession can switch between three main morphotypes: fusiform, oval, and triradiate [26]. While the fusiform morphotype is predominantly observed in natural conditions, some ecotypes show a natural tendency towards alternative morphotypes. For example, in some culture conditions, the Pt8 accession has been described to be found in triradiate morphotypes [26]; however, this morphotype seems to be unstable [27]. The Pt3 strain, derived from Pt2, was selected for its ability to grow in freshwater media, with cells that predominantly adopt an oval shape [26]. The Pt9 accession, isolated in Micronesia, exhibits fusiform morphotypes at temperatures ranging from 25 to 28 °C but is able to switch to an oval shape when cultured at temperatures between 15 and 19 °C. Similar temperature-dependent transitions are observed for Pt3 and Pt8 when the temperature decreases from 19 to 15 °C. Thus, the appearance of the oval morphotype shows the ability of *P. tricornutum* to adapt to stress conditions (salinity, temperature, or light), that differ from one strain to another [26,28]. In contrast, some accessions originally isolated from a low-salinity environment, such as Pt7, display a preference for the fusiform morphotype [26]. Recently, seven newly isolated accessions have been phenotypically characterized [29].

In addition to genetic tools, some “omics” data have also been generated: either numerous genomic [30,31,32,33], transcriptomic [27,29,34,35,36,37,38], proteomic [39,40,41,42,43,44], or metabolomic data [45,46]. “Omics” technologies are currently widely used to optimize the production of biologics in CHO cells, either in terms of titer, quality, or cell productivity [47,48]. To date, most of the “omics” data generated on *P. tricornutum* focus on how environmental conditions—such as nutrient depletion or starvation or exposure to stresses such as light/dark, toxic compounds, or predators—can affect the cell biology of a single accession compared to a control condition. Given the genetic differences and the possible variation in gene expression in response to different culture conditions, the question arises of the differences in gene expression between these accessions grown under identical conditions and growth phases. In a previous meta-analysis, we attempted to identify the best *P. tricornutum* accession for biologics production. The results of this work suggest that the Pt4, Pt3 oval, and Pt8 strains could be interesting chassis for optimizing the production of recombinant proteins and glycoproteins in *P. tricornutum* (Boulogne et al. 2024, submitted, Université de Rouen Normandie, Rouen, France, 2024 [49]); however, the limitation of this meta-analysis is the use of datasets from different RNA-Seq studies with similar but not exactly the same culture conditions. Moreover, due to the need to have the closest culture conditions, only three accessions (Pt3, Pt4, and Pt8) and cultures enriched in the three morphotypes of Pt3 (oval, triradiate, and fusiform) have been compared to Pt1 in this previous meta-analysis.

**Figure 1:**
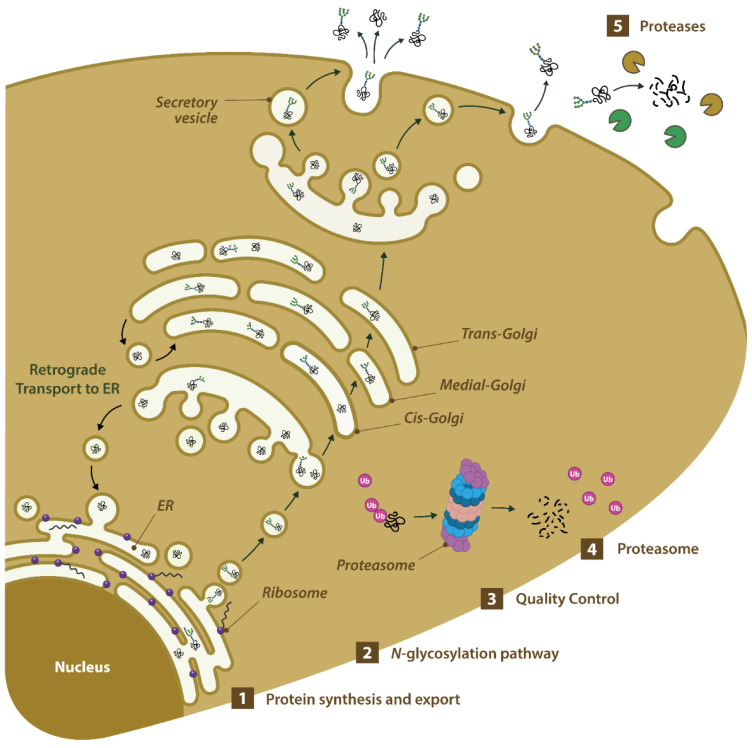
Representation of biological processes that have been shown in other organisms to have a notable influence on the production of biologics and have been studied in *P. tricornutum*.

In this context, the aim of the present study is to analyze the transcriptome of the ten most commonly used accessions of *P. tricornutum* (Pt1 to Pt10), cultivated under the exact same conditions and compared at the same exponential growth phase. This includes analyzing the expression of genes involved in biological pathways that are known to have a significant impact on the production of biologics like protein biosynthesis, protein export and secretion, *N*-glycan biosynthesis, quality control, and proteasomes or those-encoding proteases. All these pathways were compared between the *P. tricornutum* accessions, in order to propose the most suitable one(s) (Figure 1).

All these biological pathways were analyzed by comparing the ten accessions with the Pt1 accession, which was used as a reference. Indeed, Pt1 is the strain that the genome was sequenced for in 2008 [20] and represents the lab-scale model. Finally, a focus was made on the expression of specific genes whose promoters have already been used for the production of recombinant proteins in *P. tricornutum*. The results obtained provide key elements to consider when selecting a *P. tricornutum* accession strain for the production of molecules of interest.

## 2. Results and Discussion

### 2.1. Growth Kinetics

**Figure 2:**
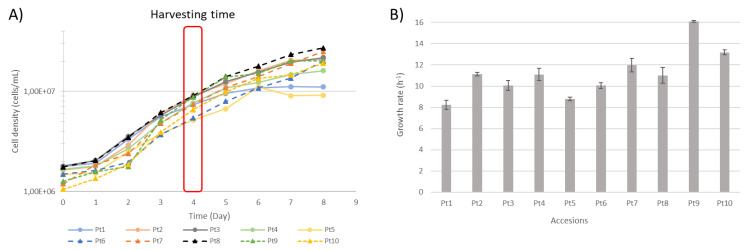
Growth parameters of the 10 most used accessions (Pt1 to Pt10). (**A**) Growth kinetics of the 10 accessions measured from day 0 (inoculation) to day 8. Cells were harvested at the same growth phase on day 4, which represents the exponential growth phase. (**B**) Growth rate of the 10 accessions, error bars indicate standard deviations of triplicate measurements.

In order to determine when the cells from the 10 accessions were in the same physiological growth state using our culture conditions, growth kinetics were performed on the 10 accessions of *P. tricornutum* (Figure 2A), allowing us to calculate and compare the growth rate (µ) (Figure 2B) and generation time (G) (Figure S1). In order to compare the gene expression of the 10 accessions in a fair manner, we decided to consider these accessions in the exponential growth phase. Based on this data set, we decided to extract mRNA from cultures at day 4, which represents the time when the 10 accessions were in the same exponential growth phase and where the growth rates are at their highest, as it is under these conditions that protein synthesis is at its highest.

Under our conditions, the highest growth rates were observed for the Pt9 and Pt10 accessions, with generation times of 1.03 and 1.26 days, respectively. In contrast, the Pt1 and Pt5 accessions grew the slowest, with generation times of 1.89 and 2.02 days, respectively. Surprisingly, another recent publication comparing the phenotypic traits of 17 accessions of *P. tricornutum* showed different results under their culture conditions [29]. Indeed, in their work, although Pt10 was one of the accessions with the fastest growth rate, Pt9 was the accession with the slowest growth rate; however, although the behavior of the accessions is different between their and our culture conditions, the growth rates are quite close for the strain Pt9, with 15.6 h^−1^ for Chaumier et al. and 16.1 h^−1^ in our experiment. When comparing the culture conditions in the two studies, the temperature and light in their and our culture conditions are quite close. Therefore, the observed differences can be attributed to either the composition of the culture media or to the inoculum size that was different. Indeed, in the study of Chaumier et al., the accessions were grown in an enriched artificial seawater (EASW) medium with an initial concentration of 1 × 10^5^ cells/mL [29], while in our study *P. tricornutum* accessions were grown in artificial seawater supplemented with Conway’s solution with an initial inoculum of 1 × 10^6^ cells/mL. In addition, it has been shown on other microalgae species such as *Chlorella* and *Scenedesmus* that the concentration of the initial inoculum dose can influence the growth behavior [50,51,52]. Moreover, a specific study conducted on *C. sorokiniana* showed that higher levels of photosynthesis-related proteins and a high turnover rate were obtained with an inoculum size of 10^6^ cells/mL. The authors of this study suggested that the culture with this inoculum size could exploit the potential for more protein synthesis in this microalgae [52]. Thus, it is tempting to hypothesize that a higher expression of endogenous protein may lead to a higher expression of recombinant proteins used as biologics. Considering its faster growth rate, we can speculate that Pt9 could present a greater synthesis of proteins in comparison to other strains, and so could be more of interest.

### 2.2. Differentially Expressed Genes Analysis

The differential expression of genes (DEGs) analysis in our culture conditions was performed with the ten ecotypes of *P. tricornutum*. Therefore, we compared the accessions from Pt2 to Pt10 with Pt1 as a reference (Figure S2). The accession Pt1 was chosen as a reference since it is the first strain that has its genome sequenced [20] and it is the most used accession so far. The volcano plots showed that there were fewer differences in Pt2 and Pt3. About 2000 genes were differentially expressed between the accessions Pt4, Pt5, Pt6, Pt8, and Pt10 compared to Pt1. Finally, the largest differences in the number of DEGs are for accessions Pt7 and Pt9, with 4219 and 5216 genes that are differentially expressed between these accessions and Pt1, respectively. The genetic diversity of the 10 accessions has been previously studied, which has allowed them to be classified into four clades, regardless of the method used for examination (internal transcribed spacer 2, amplified fragment length polymorphism, or Fixation index) [25,26,29]. Therefore, these studies show that Pt1, Pt2, Pt3, and Pt9 are clustered in genotype A; Pt4 is the only representative of genotype B; Pt5 and Pt10 belong to the genotype C; and, finally, Pt6, Pt7, and Pt8 are grouped in genotype D [25,26,29]. Although the number of DEGs in Pt2 and Pt3 is very low when compared to Pt1, Pt9—which is genetically clustered with Pt1, like Pt2 and Pt3—presents the highest number of DEGs. The observed difference in the number of DEGs may be related to epigenetic [33,53,54] and/or environmental factors such as temperature and probably not to polymorphisms, considering the few differences in terms of SNPs or INDELS within the same clade. For example, under low-temperature conditions, compared with its original environment, a phenotypic change was observed in the Pt9 accession from fusiform to oval morphotypes [28]. Therefore, the large number of DEGs observed in this work could reflect an adaptation of the diatom to the culture conditions by regulating gene expression. Overall, most of the DEGs are under-expressed when comparing the nine other accessions with Pt1 as a reference. The exceptions are Pt3 and Pt7, where the number of over- and under-expressed genes is approximately equal. In addition, the Pt9 strain shows a higher number of over-expressed genes compared to under-expressed genes. The Log2 FoldChange (Log2FC) values for under-expressed genes mostly vary between −1 (threshold) and around −11. For over-expressed genes, the Log2FC values vary between 1 (threshold) and about 6. Accessions Pt4, Pt6, and Pt10 had a greater number of genes with significantly higher differences in expression compared to the other accessions.

A GO enrichment analysis was then performed on DEGs that were specifically over- and under-expressed using all available gene set databases on ShinyGO (Table S1). Regarding down-regulated genes, the pathways found to be enriched were related to DNA (integration, metabolic processing, or nucleic acid binding) for all the accessions, except for Pt9, which was enriched for pathways related to the plasma membrane cell periphery and protein binding. Regarding up-regulated genes, no significant enrichment was found for Pt2, Pt3, and Pt10. For some accessions, enriched pathways are mainly related to transport or assimilation of elements essential for algal growth. For example, in Pt4, pathways related to nitrate were enriched, while in Pt6, four pathways related to phosphate were enriched. Such observations for Pt4 are in agreement with previous reports. Indeed, the whole genome sequencing of the 10 accessions of *P. tricornutum* revealed the presence of a higher copy number in the genome of Pt4 of the gene Phatr3_EGO2286, which is involved in nitrate assimilation [25]. In addition, a recent study highlighted a higher nitrogen uptake in Pt4 compared to Pt1 [55]. For Pt5, Pt7, and Pt8, the enriched pathways are mainly related to chlorophyll and photosynthesis. Finally, concerning Pt9, which has the more enriched pathway for up-regulated genes, a lot of enriched pathways were related to translation or peptide biosynthesis.

This first level of analysis may suggest that Pt9 could be of interest for the production of biologics, particularly for increasing production yields, considering the over-representation of genes over-expressed in pathways related to translation and peptide synthesis. To confirm this first level of analysis, we then turned our attention to biological processes that have been shown in other organisms to have a notable influence on the production of biologics (Figure 1): (1) protein synthesis and export (translation, protein export, and secretion), (2) N-glycosylation, (3) quality control, (4) proteasomes, and (5) occurrence of proteases that may accumulate in the culture media and may be responsible for proteolytic degradation of mAbs.

### 2.3. Targeted Analysis of Interesting Pathway from a Bioproduction Point of View

#### 2.3.1. Protein Synthesis: Translation and Ribosome Biogenesis

**Figure 3:**
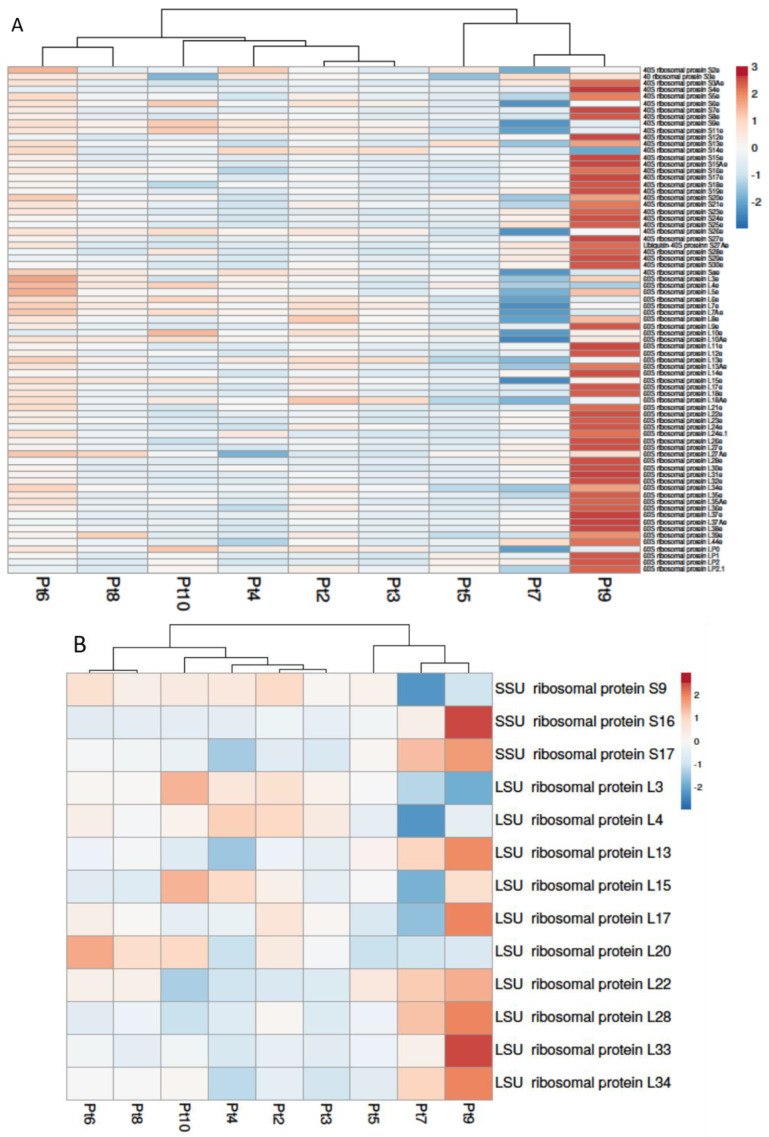
Differential expression of genes involved in ribosome pathway of *P. tricornutum*. (**A**) Expression of homologous cytosolic ribosomal genes. (**B**) Expression of homologous mitochondrial/chloroplastic ribosome genes. Under-expressed genes are shown in blue and over-expressed genes in red. Columns are clustered using correlation distance and average linkage. Gene expressions in Pt2 to Pt10 are compared to Pt1.

In eukaryotes, cytosolic ribosomes are composed of ribosomal RNA and proteins arranged in two subunits, which collaborate to guarantee accurate decoding of the mRNA sequence and the proper assembly of amino acids into a polypeptide chain [56,57]. There, we can suppose that an up-regulation of genes involved in metabolic pathways such as translation or ribosome biogenesis may result in enhanced protein synthesis and could be beneficial for improving the production yield of recombinant proteins in *P. tricornutum*. Using KEGG, 75 homologous genes encoding putative cytosolic 40S and 60S proteins were identified in the genome of *P. tricornutum* (Table S2). No significant DEGs were observed for Pt2 and Pt3. These two accessions are clustered together and are the more distant from the cluster formed by Pt7 and Pt9, which are the accessions with more DEGs (Figure 3A) in comparison to Pt1. Pt7 is the accession with the most under-expressed genes, while Pt9 is the accession with the most over-expressed genes. These observations are consistent with the previous ShinyGO analysis, which indicates that Pt9 has some enriched pathways with up-related genes related to ribosomes and translation (Table S1).

Organellar ribosomes, such as mitochondrial/chloroplastic ribosomes, are also found throughout the Eukarya and Archaeplastida kingdoms. Thirteen homologous genes encoding mitochondrial ribosomal proteins were identified in the *P. tricornutum* genome (Table S2). The clustering profile of the different accessions is the same between the expression of genes encoding cysotolic ribosomes and those encoding mitochondrial/chloroplastic ribosomes. The cluster formed by Pt2 and Pt3 is still the most distant from that formed by Pt7 and Pt9. Once again, Pt9 is the strain with the most over-expressed genes, with 8 out of 13 over-expressed genes (Figure 3B). Based on these initial observations, we can suggest that Pt9 may be a promising candidate for improving the synthesis and production of recombinant proteins. Nevertheless, further studies, such as proteomic analysis of this accession under these conditions, would be necessary in the future to ascertain whether the observed up-regulation of over 70% of the genes involved in ribosome biogenesis will result in an enhanced production yield of proteins.

#### 2.3.2. Protein Export

To achieve higher production yields of biologics, efficient protein expression is undoubtedly a crucial factor but not the only critical point. Indeed, in some systems such as plants, processing steps like cell harvesting, lysis, and purification are time-consuming and contribute to higher production costs [58]. Thus, the optimal expression system should not only facilitate high protein expression but also involve efficient protein secretion, reducing costly downstream processing steps that represent approximately 84% of the total cost in expression systems like plants [58]. With regards to mammalian cells, microalgae are capable of secreting proteins into the culture medium [43,59]. The secretion of biologics has also been reported in two species of microalgae [17,18,60,61], including *P. tricornutum*, for which the production of two secreted anti-viral mAbs has been described [17,18].

**Figure 4:**
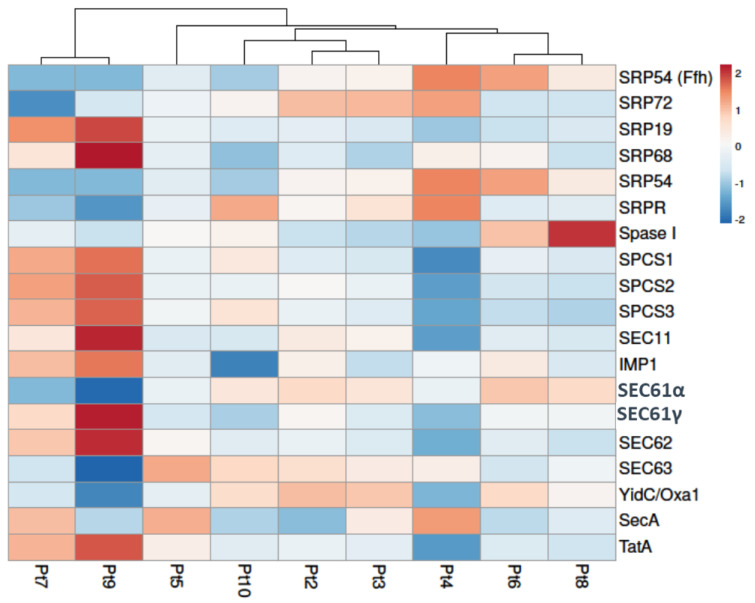
Differential expression of genes involved in protein export and secretion pathways of *P. tricornutum*. Under-expressed genes are shown in blue and over-expressed genes in red. Columns are clustered using correlation distance and average linkage. Genes in Pt2 to Pt10 accessions are compared to Pt1 used as a reference in this study.

Protein export, the active transport of the proteins outside the cell, occurs through two main mechanisms: the co- and post-translational delivery mechanisms. During the co-translational mechanism, the nascent polypeptides are transported to the export membrane along with the ribosome during protein synthesis. In contrast, in the post-translational mechanism, proteins are delivered to the outside of the cell after the synthesis is completed. Orthologues for molecular actors involved in these two mechanisms have been identified in the genome of *P. tricornutum* (Table S3). Their expression profiles were studied and compared in the course of the present work for the 10 accessions. It was observed that genes involved in the protein export are differentially expressed mostly in Pt4, Pt7, and Pt9. It is somewhat surprising that the Pt4 accession was not hierarchically closer to the cluster formed by the Pt7 and Pt9 accessions. The accession with the greatest number of over-expressed genes is Pt9. In contrast, Pt4 has the greatest number of down-regulated genes (Figure 4).

In the co-translational mechanism, peptide chains are first synthesized by the ribosome. Signal recognition particles (SRPs) simultaneously recognize and bind to the signal peptide and the SRP receptor (SRPR), forming a complex with the ribosome on the endoplasmic reticulum (ER) membrane. Two SRPs (SRP19 and SRP68) are over-expressed in Pt9, while in Pt7, SRP72 is under-expressed in these two accessions. This complex allows the peptide to enter the ER lumen. At the same time, signal peptides are removed by signal peptidases. According to the KEGG database, orthologues of both eukaryotic (SPCS1, SPCS2, and SPCS3) and prokaryotic (Spase I) signal peptidases are described in *P. tricornutum* (Table S3). These genes are under-expressed in Pt4, while all of them are over-expressed in Pt9.

Eukaryotic, prokaryotic, and mitochondrial orthologues of important actors of the post-translational mechanism are also described in *P. tricornutum* (Table S3). Three components of the eukaryotic translocation channel are differentially expressed in Pt9, Sec61α is under-expressed, while Sec61γ and Sec62 are over-expressed. A homolog of the bacterial chaperone YidC/Oxa1 is under-expressed in Pt4, Pt7, and Pt9. Another protein transport system, the twin-arginine translocation (Tat) pathway, is responsible for the transport of folded proteins in bacteria, archaea, and chloroplasts. TatA, one of the membrane proteins involved in this pathway, is under-expressed in Pt4 and over-expressed in Pt9.

Interestingly, the results for Pt4 diverge significantly from those of our previous meta-analysis study of the same genes, in which Pt4 exhibited 12 genes over-expressed (Boulogne et al., submitted [49]). This discrepancy may be attributed to the difference in culture conditions used to generate the datasets selected for the meta-analysis and the conditions selected for this present work. Indeed, in the meta-analysis, cells were harvested at 6 days of culture following 2 days in the dark [36]. In contrast, the present work examined mRNA expression extracted after 4 days of culture under a light/dark cycle of 16/8 h. The overexpression of genes involved in the protein export pathway in Pt9 may also be linked to a possible shift in morphotype from fusiform to oval of this accession due to the temperature of 19 °C used in this study [26]. Studies have demonstrated a higher and faster expression and secretion of proteins in oval cell cultures in comparison to triradiate and fusiform cell cultures of the same accession [43,62]. The overexpression of genes involved in the co-translational mechanism, in which ribosomes are strongly involved, appears to be consistent with the GO enrichment analysis that indicated an overexpression of genes involved in ribosome biogenesis in Pt9.

This new level of analysis focused on protein export also indicated the potential of the Pt9 strain as a means to enhance protein production.

#### 2.3.3. N-Glycosylation

Most biologics are glycoproteins—meaning proteins bearing glycans, either *O*- or *N*-linked—that can significantly impact their biological activity and immunogenicity [1,63]. Therefore, special attention must be paid to the glycosylation processing that is required when choosing an organism for the production of glycosylated biologics. Currently, there is no known information regarding the *O*-glycosylation processing in *P. tricornutum*. In contrast, the addition of *N*-glycans onto proteins occurs like the other eukaryotes, in the secretory system as the proteins progress through the ER and the Golgi apparatus to their final compartment [64] (Figure 1). The recombinant mAbs produced in Pt4 have been shown to be predominantly glycosylated with mammalian-like oligomannosides. No complex-type *N*-glycans or immunogenic glycoepitopes were identified on the produced mAbs [65], although *P. tricornutum* possesses the putative glycoenzymes that are involved in the synthesis of such complex *N*-glycans [66].

**Figure 5:**
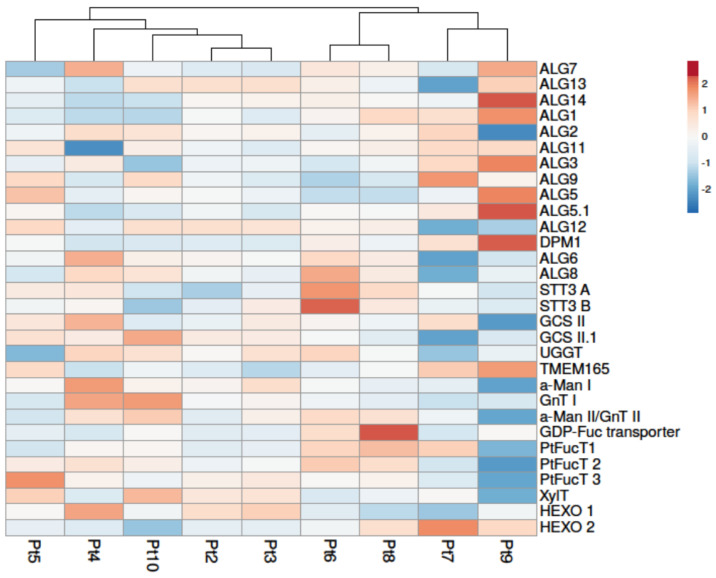
Differential expression of genes involved in the protein *N*-glycosylation biosynthesis pathway of *P. tricornutum*. Under-expressed genes are shown in blue and over-expressed genes in red. Columns are clustered using correlation distance and average linkage. Genes in Pt2 to Pt10 are compared to Pt1.

With regards to the overall genes involved in the *N*-glycosylation pathway of *P. tricornutum* (Table S4), it is observed that there are more differences in accessions Pt7 and Pt9 compared to the other accessions (Figure 5). In fact, Pt7 is the accession with the greatest number of under-expressed genes compared to Pt1 and Pt9 is the one with the greatest number of over-expressed genes. No DEGs related to the *N*-glycosylation pathway were identified in Pt2 or Pt3. Once again, the hierarchical cluster formed by those accessions is more distant from the one constituted by Pt7 and Pt9.

The synthesis of *N*-glycans starts in the ER with the synthesis of a precursor called the lipid-linked oligosaccharide (LLO). This precursor is synthesized by the action of ALGs (asparagine-linked glycosylation) enzymes that use nucleotide sugars and their specific acceptors to elongate the LLO [67,68]. Among the 13 genes involved in the synthesis of the LLO, some are differentially expressed in different accessions. The most notable differences are observed in Pt7 and in Pt9 which present, respectively, two significant down-regulated genes and six significant up-regulated genes. These results suggest an increased synthesis of the LLO in Pt9.

The synthesized LLO precursor is subsequently transferred *en bloc* to the asparagine at the consensus *N*-glycosylation site (Asn-X-Thr/Ser/Cys) by an enzyme complex known as oligosaccharyltransferase (OST) [69,70]. Two genes encoding putative catalytic subunits (SST3A and SST3B) of the OST have been identified in the *P. tricornutum* genome. In our conditions, the only differential expression observed concerned the gene Phatr3_J55198 encoding for the catalytic subunit SST3B, which was found to be differentially expressed in Pt6 and Pt10. The maturation of the *N*-glycan attached to the glycoprotein is then carried out by two ER-resident enzymes: glucosidase II (GCS II) and UDP-glucose—glycoprotein glycosyltransferase (UGGT). Once correctly folded, the glycoproteins leave the ER to reach the Golgi apparatus, where maturation steps involve a variety of glycoenzymes that result in species-specific complex *N*-glycan structures [71].

Regarding the *N*-glycosylation steps that occur within the Golgi apparatus, our attention was focused on the expression of specific genes encoding for *N*-acetylglucosaminyltransferase I (GnT I), GDP-Fucose (GDP-Fuc) transporters, fucosyltransferases (PtFucT), xylosyltransferases (XylT), and *N*-acetylhexosaminidases (HEXO) (Figure 5) as they are key glycoenzymes for the synthesis of complex-type *N*-glycans, which are critical quality attributes for the activity and the safety of biopharmaceuticals. GnT I is a key enzyme for the initiation of the synthesis of complex-type *N*-glycans [72]. It is over-expressed in Pt4 and Pt10 but under-expressed in Pt5, Pt7, and Pt9. The over-expression of this enzyme could favor the selection of Pt4 and Pt10 accessions when complex-type *N*-glycans are required on the recombinant glycoproteins. The over-expression of GnT I has already been observed in the Pt4 accession in our meta-analysis (Boulogne et al., submitted [49]), although the culture conditions used were different from our current conditions, particularly in terms of pre-harvest culture time and exposure to darkness. However, the characterization of the anti-VHB mAbs produced in Pt4 showed that only oligomannosidic *N*-glycans but not complex-type *N*-glycans were detected [65]. The absence of complex-type *N*-glycans on mAbs could be explained by the nature of this protein, which has its *N*-glycosylation sites embedded, limiting the access of glycosyltransferases to the *N*-glycans, or by the culture conditions used for the production of such mAbs, which do not allow the over-expression of GnT I and the other glycoenzymes involved in the synthesis of complex-type *N*-glycans in *P. tricornutum*.

Core α-(1,3)-fucose and core β(1,2)-xylose glycoepitopes present on plant *N*-glycans have been reported to be immunogenic in humans [73,74]. Moreover, it is well-known that the core fucosylation can affect the biological function of mAbs. A recent study highlighted an increase in α-(1,3)-fucosylation on endogenous glycoproteins of *P. tricornutum* when PtFucT1 is over-expressed [75]. The GDP-Fuc transporter plays a critical role in the fucosylation by allowing the import of GDP-Fuc into the Golgi apparatus where it is the substrate of FucT. Therefore, specific attention was paid to the 5 genes related to core fucosylation and xylosylation in *P. tricornutum*. Among them, only the genes encoding the GDP-Fuc transporter and PtFucT1 were significantly over-expressed and only in the Pt8 accession. Finally, putative *N*-acetylhexosaminidases that can lead to truncated *N*-glycan structures called paucimannoses that lack terminal GlcNAc residue have been identified in *P. tricornutum* [65,66]. Two genes encoding *N*-acetylhexosaminidases are predicted in *P. tricornutum*. The gene Phatr3_J45073 (HEXO1) is under-expressed in Pt7 and Pt8 but the gene Phatr3_J49563 (HEXO2) is over-expressed in Pt7. However, the activity of these enzymes has not yet been demonstrated in *P. tricornutum*.

Based on all this information, the Pt4, Pt9, and Pt10 accessions appear to be slightly more interesting in terms of *N*-glycosylation to produce biologics. Indeed, if the cell is to produce more recombinant proteins, the *N*-glycosylation processing must be able to keep up to ensure that the synthesized proteins are correctly glycosylated. In this case, Pt9 is of interest because it has six genes that are over-expressed in the ER. Pt4 and Pt10 are interesting because of the over-expression of the GnT I that could lead to the presence of more complex type *N*-glycans. However, further glycomic studies need to be performed to compare the protein *N*-glycosylation of the 10 accessions and to decipher whether or not the over-expression of these genes can impact *N*-glycan structures.

#### 2.3.4. Quality Control and Proteasomes

**Figure 6:**
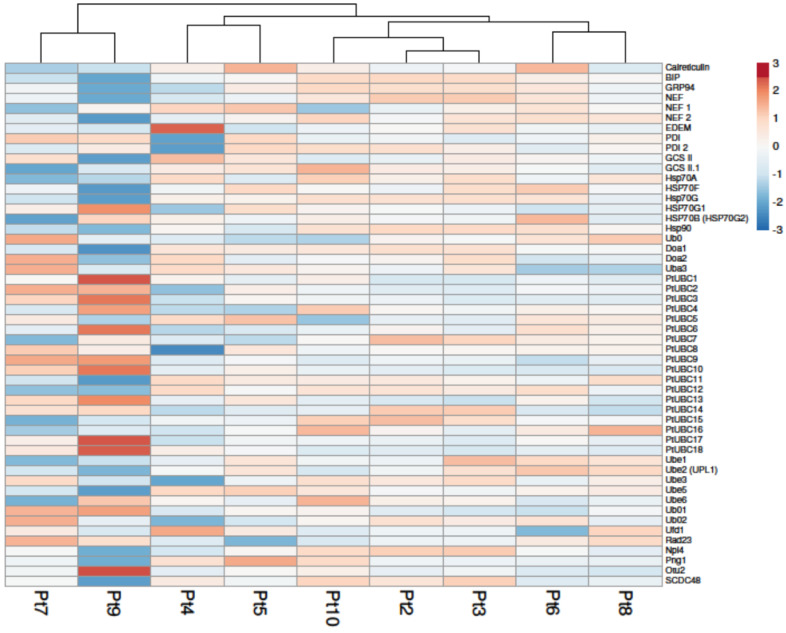
Differential expression of genes involved in protein quality control pathway of *P. tricornutum*. Under-expressed genes are shown in blue and over-expressed genes in red. Columns are clustered using correlation distance and average linkage. Expression of genes in Pt2 to Pt10 was compared to that of Pt1, used as a reference accession.

Proteins undergo a quality control process within the ER before being accumulated into various intracellular compartments or released into the extracellular media. This process involves interactions with multiple quality control factors to ensure the release of properly folded and functional proteins. In particular, nascent proteins interact with chaperones such as calreticulin, calnexin, and heat shock proteins (HSPs), including BiP (binding immunoglobulin protein) and GRPs (glucose-related proteins) [76,77]. The analysis of the DEGs involved in the quality control (QC) in *P. tricornutum* revealed that most of the differentially expressed genes tended to be under-expressed (Figure 6). More differentially expressed genes are observed for Pt4, Pt7, and Pt9, while no differential expression of genes is observed in Pt2 and Pt3. Regarding luminal chaperones (Table S5), the gene Phatr3_J16786 that encodes the protein GRP94—a chaperone specialized for protein folding in the ER—is down-regulated in Pt4 and Pt9; however, GRP-94 seems to be essential primarily for metazoans but not for unicellular organisms such as mammalian cell culture [78]. The gene Phatr3_EG02643 encoding BiP is also down-regulated in Pt4, Pt7, and Pt9. Recently, it has been highlighted that when the basal expression level of BiP in host cell lines is low, it facilitates the generation of recombinant CHO cell lines (rCHO) that give a higher true positive rate during random integration-based pool selection [79]. However, some studies have shown in cell lines producing mAb that the expression level of BiP and mAb production were correlated [79,80,81]. For *N*-glycosylated proteins, recognition of the oligosaccharide on their surface occurs by the ER luminal enzymes glucosidases II (GCSII), which sequentially cleave terminal glucose residues. Prior to the removal of the third glucose by the GCSII, chaperone proteins assess the correct folding of glycoproteins, and direct properly folded proteins to the secretory pathway. Misfolded proteins, with all three glucose residues removed, are identified by UDP-glucose/glycoprotein glucosyltransferase (UGGT), leading to the addition of glucose residues and subsequent re-engagement with chaperones [77,78,79]. Abiotic or biotic stresses (Difficult To Express (DTE) protein production or environmental stresses) can provoke a deregulation of the homeostasis in the ER leading to an accumulation of misfolded/unfolded proteins in the ER and to the activation of the unfolding protein response (UPR) or ER-associated degradation (ERAD) pathway [79,80]. In mammals, it has been shown that the production of DTE proteins such as mAbs can lead to the activation of the UPR pathway to restore ER homeostasis. Among all the mechanisms involved in the ERAD pathway, ubiquitination plays a crucial role by affecting the stability of the protein. Ubiquitin-related enzymes have been shown to be important for stress regulation in mammals [82,83,84], plants [85,86,87], and also in *P. tricornutum* [88]. Regarding the genes involved in the ERAD pathway, most of the genes are also down-regulated. Recently, 18 UBC genes (PtUBC1 to PtUBC18) were identified in *P. tricornutum* [88]. Among these genes, half of them were differentially expressed in Pt9, with a majority of up-regulated genes. The up-regulation of genes related to ubiquitination could suggest that the ER is under stress and that the cell responds accordingly. As mentioned above, the Pt9 accession seems to adopt an oval morphotype (stress-related) at temperatures between 15 and 19 °C, the latter being the temperature used for the cultures analyzed in this study. This could explain why more ERAD-related genes are up-regulated in this accession.

**Figure 7:**
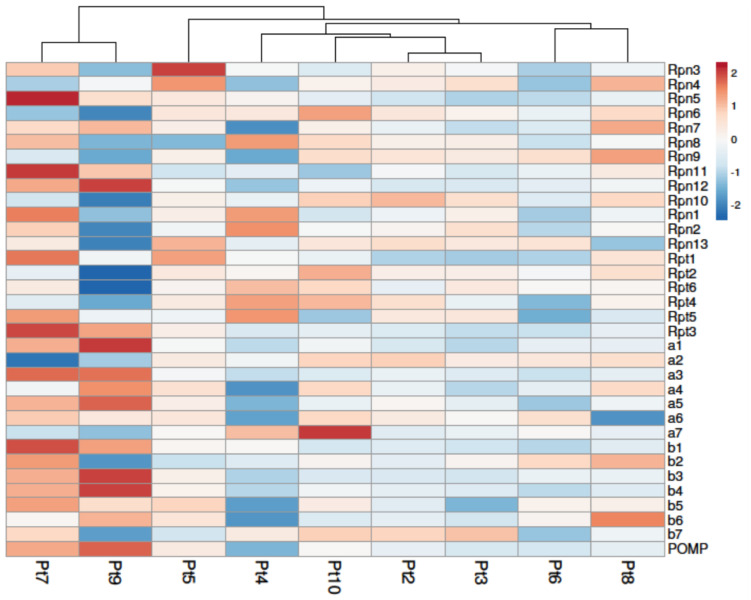
Differential expression of genes involved in the proteasome pathway of *P. tricornutum*. Under-expressed genes are shown in blue and over-expressed genes in red. Columns are clustered using correlation distance and average linkage. Expression of genes in Pt2 to Pt10 was compared to that of Pt1, used as a reference accession.

Taking into consideration the elements provided by the study of the regulation of genes related to QC and proteasomes (Figure 6 and Figure 7), we can suggest that the Pt4, Pt7, and Pt9 accessions could be of interest for the production of biologics. Indeed, the down-regulation of genes related to these pathways could indicate that these accessions would have a better capacity to produce correctly folded proteins and would have less need to recruit the various players involved in the regulation of ER homeostasis. Thus, under more stressful conditions, such as the production of DTE proteins, these accessions would have greater leeway to regulate ER homeostasis. It would be interesting in future studies to compare these different accessions—genetically transformed for the production of biologics—to monitor the expression of QC-related genes and to see if mAb production could be correlated with the expression of chaperone proteins, as it has been previously shown in mammalian cells [79,80,81].

#### 2.3.5. Proteases

The use of microalgae as a cost-effective production platform on an industrial scale is currently limited by their low production yields, which are lower than those obtained with optimized CHO cells [18,89]. These lower yields, reflecting a low accumulation of secreted proteins in the culture medium, could be partly attributed to the potential proteolytic degradation of mAbs in the culture medium. Indeed, while host cell proteases play an essential role in catalytic and metabolic pathways [90], their presence in the culture media can lead to the degradation of the product of interest [91]. The presence of proteases in the culture media can result from either host cell secretion and/or cell lysis. The negative impact of proteolytic degradation on the yield of recombinant proteins accumulated in culture media has already been demonstrated in CHO cells [91,92] and in plant systems [93]. In addition, the rigorous purification steps of biologics do not always allow for complete removal of residual protease activity from CHO cell lines [94]. To date, nothing is known regarding the proteolytic degradation of recombinant proteins accumulated in *P. tricornutum*’s culture medium. Two recent studies on the secretome of *P. tricornutum* indicate the presence of several putative proteases in the culture medium of this microalgae [43,95].

**Figure 8:**
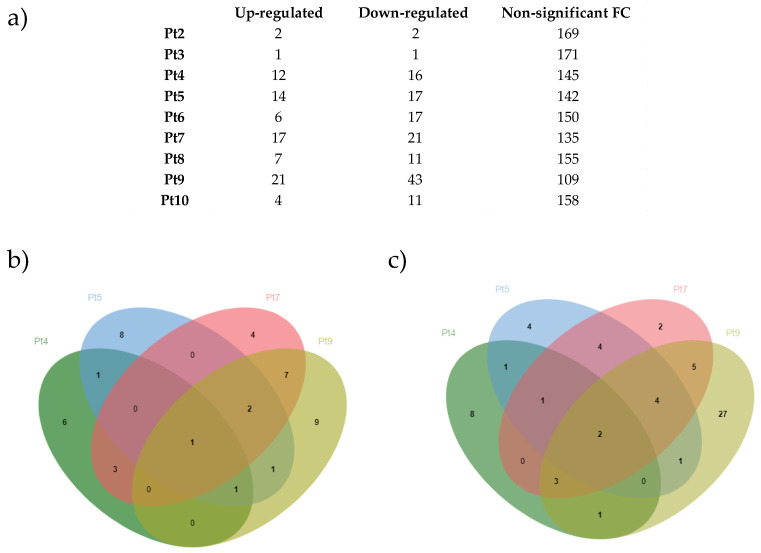
Differential expression of genes encoding proteases identified in *P. tricornutum*. (**a**) Number of differentially expressed proteases or with a non-significant fold change in Pt2 to Pt10 accessions compared to Pt1. Genes involved in protease activities were obtained from UniprotKB (keyword: KW-064 Protease) and from DiatomicBase. Up-regulated genes have a significant fold change > 2 (i.e., log2FC > 1), *p* < 0.05; down-regulated genes have a significant fold change <0.5 (i.e., −1 < log2FC), *p*-value < 0.05. (**b**) Venn diagram of up-regulated proteases in Pt4 (dark green), Pt5 (blue), Pt7 (red), and Pt9 (light green). (**c**) Venn diagram of down-regulated proteases in Pt4 (dark green), Pt5 (blue), Pt7 (red), and Pt9 (light green).

**Figure 9:**
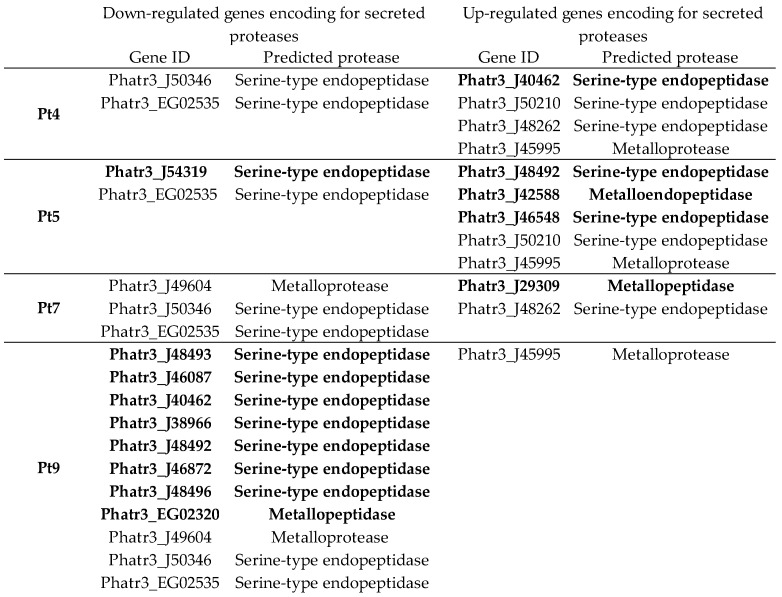
Genes of secreted proteases (according to [43,95]) up- and down-regulated found in Pt4, Pt5, Pt7, and Pt9 (compared to Pt1). Genes encoding proteases specifically found in the corresponding accession are indicated for further information in bold.

One hundred and seventy-three putative proteases were identified in *P. tricornutum*. For all accessions, few genes encoding putative proteases are significantly differentially expressed compared with Pt1 (Figure 8a). The largest differences were observed for Pt4, Pt5, Pt7, and Pt9. Due to their larger number of DEGs, we decided to focus only on these four accessions for the remaining analysis regarding proteases (Figure 9). Firstly, we were interested in the expression of proteases that were specifically over-expressed (Figure 8b) or under-expressed (Figure 8c) in each accession.

Among these specifically over- or under-expressed proteases, we used recent data generated on the *P. tricornutum* secretome [43,95] to investigate whether any of them could be secreted into the culture medium. Looking at the down-regulated genes, some are specifically expressed in one accession, while others like the Phatr3_EG02535 gene are under-expressed in all four accessions. Among up-regulated genes, similar observations can be noticed. The type of secreted proteases whose genes are differentially expressed are predominantly serine-type endopeptidases, followed by metalloproteases or metallopeptidases. Serine-type and metalloproteases have already been shown to be problematic in the context of recombinant protein production in CHO cells [96,97,98,99]. Released into the culture medium as HCPs, these proteases can lead to protein degradation [96,97,98,99]. Furthermore, their elimination during purification steps is not always guaranteed. In fact, some HCPs such as the serine protease HTRA1 have been shown to co-elute with mAbs using protein A chromatography [100,101,102].

**Figure 10:**
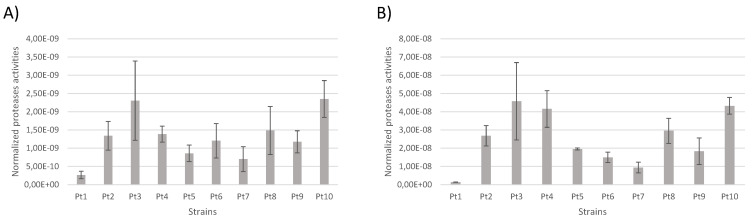
Protease activities normalized per cell of the 10 accessions of *P. tricornutum*. (**A**) Activities of intracellular serine-type, cysteine-type, and acidic proteases of the 10 accessions of *P. tricornutum*. (**B**) Activities of secreted serine-type, cysteine-type, and acidic proteases of the 10 accessions of *P. tricornutum*. Assays were performed on three biological replicates.

In addition, biochemical assays of protease activities were carried out on the 10 *P. tricornutum* accessions. The results show that protease activities are lower inside the cells (Figure 10A) than in the extracellular medium (Figure 10B). Activity profiles between strains are fairly similar when comparing the intracellular protease activities to the ones present in the culture medium. In contrast to the analysis of DEGs, the protease activities of all accessions are stronger than the one observed for Pt1. The highest activities are observed in Pt3 and Pt10 accessions, even though they were among those with fewer over-expressed protease-encoding genes in comparison to Pt1; lower activities are observed in the Pt1 and Pt7 accessions. The discrepancy in results between differential gene expression and biochemical characterization of protease activities can be attributed to the fact that the assay kit is targeting different types of proteases that are serine-type, cysteine-type, and acid-type. Moreover, during our analysis of DEGs, we only analyzed genes identified as encoding for proteases. However, it is important to remember that approximately 25–30% of *P. tricornutum* genes are not well-characterized and are still annotated to encode for “predicted proteins”. Therefore, one can hypothesize that additional protease activities are still to be discovered in *P. tricornutum*.

If we look specifically at the genes encoding secreted proteases, the Pt7 and especially the Pt9 accession could be of interest for the production of biologics because they have more related genes down-regulated than up-regulated. In line with this, a better amount of recombinant proteins can be expected to accumulate in the culture media of such accessions. The biochemical characterization of protease activities tends to confirm the DEGs analysis. Indeed, Pt1, Pt6, Pt7, and Pt9 present lesser protease activities in the culture media in comparison to the other strains.

#### 2.3.6. Genetic Tools

**Figure 11:**
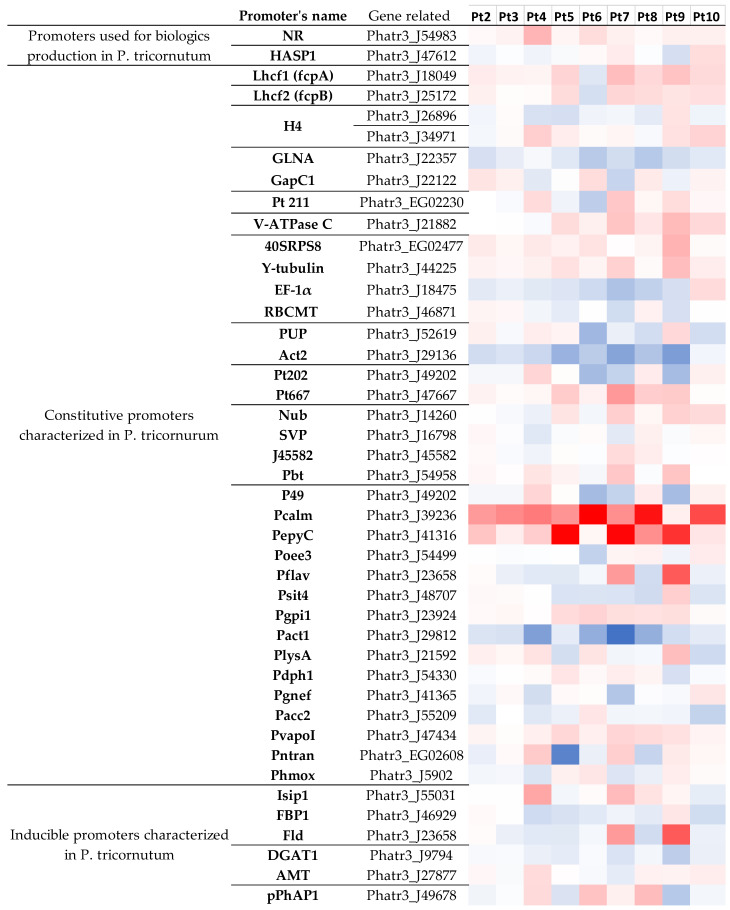
Differential gene expression of genes whose promoters have been characterized in *P. tricornutum*. Promoters that have already been used to produce biologics in *P. tricornutum*, as well as constitutive and inducible promoters that have already been characterized, are presented. Under-expressed genes are shown in blue and over-expressed genes in red. The more intense the color, the stronger the differential expression is. Genes in Pt2 to Pt10 are compared to Pt1.

The controlled expression of foreign genetic material is a fundamental requirement to produce recombinant proteins such as biologics. In this context, the identification of genetic elements that ensure a strong and consistent expression of genes encoding the protein of interest is crucial. A lot of work has already been published regarding the design and enhancement of the CHO expression system, including the characterization of elements that improve the yield of recombinant protein production [103,104,105,106,107]. For now, the available information on *P. tricornutum* is limited to elements such as promoters and 5′ untranslated regions (UTR) sequences (Figure 11) or terminators and 3′UTR elements [24,108,109]. With regard to this latter aspect, we postulated that if a gene exhibits a high level of expression, this could potentially indicate that the corresponding promoter is a strong promoter, and therefore, a useful tool for recombinant protein production. This hypothesis has already been tested by Garza and coworkers, who examined the promoter upstream of the gene Phatr3_J49202, which was “consistently observed to be one of the most highly expressed genes in transcriptomic datasets from *P. tricornutum*” [109]. The results indicate that this promoter can drive a higher level of activity in comparison to the promoter currently utilized in the Pt1 accession, such as the fcpb, H4, or NR promoters [109]. However, in our conditions, the expression of this gene was found to be under-expressed in Pt6, Pt7, and Pt9. Thus, it seems that the strength of the promoter must be tested in accordance with the selected accession in order to identify the optimal tandem promoter/accession combination. It would be of interest in further studies to ascertain whether the expression of a given protein differs when this promoter is tested in these accessions in comparison to Pt1.

Firstly, a survey was conducted on the promoters used for the production of biologics in *P. tricornutum*. To date, only a limited number of biologics have been produced in this microalga. Two antibodies directed against lethal viral epitopes were produced with each chain under the control of the *nitrate reductase* (NR) promoter [16,17,18], and the RBD of the SARS-CoV-2 was produced under the control of the HASP1 promoter [19,110]. With regard to these genes, the only differential expression observed concerns the gene Phatr3_J54983, which encodes for nitrate reductase that is over-expressed in Pt4 (Figure 11). This result seems to be consistent with the enrichment analysis, which showed an over-representation of over-expressed genes in nitrogen-related pathways in Pt4.

Then, the gene expression driven by other promoters already characterized in *P. tricornutum*—which were either constitutive [24,108,109,111,112,113,114,115,116,117,118] or inducible [117,119,120,121]—were analyzed as these promoters represent useful tools for the production of recombinant proteins. Most differences were observed for Pt7 and Pt9 (Figure 11). These two accessions are also the ones with the most up-regulated genes, with seven and nine up-regulated genes for Pt7 and Pt9, respectively, while Pt6 has the most down-regulated genes. Some genes appear to be more universally differentially expressed between different accessions. This is exemplified by Phatr3_J39236 which is over-expressed in all accessions, except Pt9, but also for Phatr3_J29136 which is down-regulated in 6 accessions. Consequently, it can be proposed that the promotor of the gene Phatr3_J39236 (Pcalm) could be employed as a versatile promote as its overexpression, is observed in most of the accessions, thus, it represents an interesting promoter to enhance the production of the recombinant protein of interest in *P. tricornutum*. The relevance of inducible promoters for the production of biologics may be questioned, particularly when the biologics are not lethal for the host cell (in such cases, it is necessary to use such promoter to produce the product of interest without interfering with the growth of the cell). This is especially true when the promoters rely on the starvation of a key element, such as phosphate for the alkaline phosphatase promoter (pPhAP1) [121] or the HASP1 promoter [19]. Indeed, certain elements such as phosphorus or nitrogen have been demonstrated to be essential for cell growth and fitness. Furthermore, when deprived of these elements, it has been observed that *P. tricornutum* tends to redirect these metabolic pathways towards lipid production as a stress regulation mechanism, thus limiting protein synthesis [35,41,44,45,122,123]. Nevertheless, despite the demonstrated efficacy of these methods for the production of recombinant proteins, the question remains as to their robustness when applied at an industrial scale.

A comprehensive analysis of the differentially expressed genes (DEGs) revealed the overexpression of several genes in multiple accessions. Among these, the gene Phatr3_J339237 is one of the top over-expressed genes in the accessions Pt2, Pt3, Pt5, Pt6, Pt8, and Pt10. With regards to genetic elements, it appears that no strain is of particular interest in comparison with another and that no promoter among those tested is truly universal. In this case, it would be advisable to adapt the choice of promoter to the strain used for the production of proteins of interest. Future studies may benefit from evaluating the efficacy of this promoter for recombinant protein production in these accessions. Nevertheless, it is also important to consider the combination of promoter and terminator, as some studies have demonstrated that protein expression can vary depending on the terminator used in combination with a given promoter [24,108,109].

## 3. Materials and Methods

### 3.1. Cell Culture and Growth Conditions

The ten *P. tricornutum* accessions Pt1 (CCMP 2561), Pt2 (CCMP 2557), Pt3 (CCMP 2558), Pt4 (CCMP 2559), Pt5 (CCMP 630), Pt6 (CCMP 631), Pt7 (CCMP 1327), Pt8 (CCMP 2560), Pt9 (CCMP 633), and Pt10 (CCMP 2928) used in this study were purchased from Bigelow Laboratory (National Center for Marine Algae and Microbiota). Cells were cultured at 19 °C with a 16 h/8 h light–dark cycle in 100% artificial seawater (33.3 g·L^−1^ of sea salt (Instant Ocean^®^, Blacksburg, VA, USA) filtered through 0.45 µm filters and autoclaved. The sterilized culture medium was supplemented by 1 mL·L^−1^ of adjusted Conway medium (Na_2_EDTA·2H_2_O: 45 g·L^−1^; NaNO_3_: 1000 g·L^−1^; H_3_BO_3_: 33.6 g·L^−1^; NaH_2_PO_4_: 20 g·L^−1^; FeCl_3_: 0.768 g·L^−1^; ZnCl_2_: 21 mg·L^−1^; CoCl_2_, 6H_2_O: 20 mg·L^−1^; (NH_4_)_6_Mo_7_O_24_, 4H_2_O: 9 mg·L^−1^; CuSO_4_, 5H_2_O: 20 mg·L^−1^; MnCl_2_,4H_2_O: 360 mg·L^−1^; B1 vitamin: 200 mg·L^−1^; B12 vitamin: 10 mg·L^−1^).

### 3.2. Growth Curves

The ten accessions were adapted over several months using the same culture conditions. Then, the cultures were inoculated at an initial concentration of around 1 × 10^6^ cells per mL to perform the experiments. To generate the growth curves, samples were taken every 24 h for 8 days and cell density was measured using the Countess 3 Cell Counter according to the instructions of the manufacturer (ThermoFisher Scientific^®^, Carlsbad, CA, USA). Growth rates were determined with Excel using the slope of the exponential growth phase, corresponding to the following formula:

$$\mathrm{Growth}\;\mathrm{rate}\;µ=\frac{log2N2-log2N1}{t2-t1}$$

### 3.3. Preparation of RNA Samples

For each accession, 150 mL of cell culture was inoculated at 1 × 10^6^ cells.mL^−1^ in triplicate. Cells were harvested the fourth day after inoculation by centrifugation at 5000× *g* for 5 min at room temperature. The total cell pellet was resuspended in 1 mL of Nucleozol^®^ (Macherey-Nagel, Hoerdt, France). Total RNA was extracted using lysing beads (E-matrix lysing tubes, MP Biomedicals^®^, Fisher Scientific, Illkirch, France) and grounded for 4 cycles of 30 s at 6.5 m·s^−1^ in a FastPrep-24^TM^ homogenizer (MP Biomedicals^®^). Between each run, lysing tubes were placed on ice. Lysis tubes were incubated at room temperature (RT) for 5 min, then centrifuged at 12,000× *g* for 5 min, and the supernatant was collected in RNAse-free collection tubes. A total of 400 µL of RNAse-free water was added and tubes were mixed by inversion. Tubes were incubated at RT for 15 min, then centrifugated at 16,000× *g* for 15 min at 4 °C. The aqueous phases were collected and pooled in a new RNAse-free tube, and 900 µL of ultrapure ethanol was added. Samples were homogenized by pipetting and 650 µL of the samples was loaded onto a NucleoSpin RNAPlus Column (Macherey-Nagel^®^). Tubes were centrifugated at 11,000× *g* for 30 s. These steps were repeated until the entire sample had passed through the column. From this stage onwards, the washing and elution steps were carried out using the Nucleospin RNA Plus kit (Macherey-Nagel^®^) according to the manufacturer’s instructions. The DNAse treatment was performed following the two-step incubation procedure from “rigorous DNAse treatment” of the Turbo DNA-free^TM^ kit (Invitrogen, Carlsbad, CA, USA). Total RNA concentration was measured by spectrophotometry using the NanoDrop^TM^ One (ThermoFisher Scientific). The 30 RNA samples were diluted to a maximum of 3 ng·µL^−1^ and heated at 70 °C for 2 min to minimize RNA secondary structures. A total of 1 µL of each sample was used to assess the quality of the RNAs, using the Agilent 2100 Bioanalyzer system (Agilent, Santa Clara, CA, USA) and RNA 6000 Pico Kit (Agilent).

### 3.4. Generation of cDNA Libraries and Quality Control of Reads

After quantity and quality checks, RNA sequencing was performed at the MGX facility (Montpellier, France). Libraries were prepared using the Stranded mRNA Prep Ligation kit (Illumina, San Diego, CA, USA) according to the manufacturer’s instructions. Briefly, polyadenylated RNAs were selected using oligo-dT magnetic beads from 1000 ng of total RNA. The polyA+ RNAs were fragmented and reverse-transcribed using random hexamers, Super Script II (Thermo Fisher Scientific, Carlsbad, CA, USA), and actinomycin D. Deoxy-TTP was replaced by dUTP during the second-strand synthesis to prevent its amplification by PCR. Double-stranded cDNAs were adenylated at their 3’ ends and ligated to Illumina’s adapters containing unique dual indexes (UDI). Ligated cDNAs were PCR amplified (11 cycles) and the PCR products were purified using AMPure XP Beads (Beckman Coulter Genomics, Brea, CA, USA). The size distribution of the resulting libraries was monitored using a Fragment Analyzer (Agilent Technologies, Santa Clara, CA, USA) and the libraries were quantified using the KAPA Library quantification kit (Roche, Basel, Switzerland). Library preparation was realized on three biological replicates for each *P. tricornutum*’s accession. The libraries were denatured with NaOH, neutralized with Tris-HCl, and diluted to 150 pM. Clustering and sequencing were performed on a NovaSeq 6000 (Illumina, San Diego, CA, USA) using the paired-end 2 × 150 nt protocol on one lane of an S4 flow cell. Image analysis and base calling were realized in real-time using the NovaSeq Control Software and the Real-Time Analysis 3 software, respectively (Illumina, San Diego, CA, USA). Demultiplexing and FASTQ file generation were carried out using Illumina’s bcl2fastq software (v2.20.0.422). The quality control of raw data and demultiplexed reads were assessed, respectively, using Illumina’s Sequencing Analysis Viewer (SAV) software and the FastQC (v.0.11.9, Illumina Inc., San Diego, CA, USA) software from the Babraham Institute. Contaminant screening was performed using FastQ Screen (v0.14.0, Babraham Institute). The quality control highlighted that the large majority of the sequence was of very good quality with a Phred score of 33, and no contaminants were detected. The percentage of sequences passing the Illumina PF (Purity Filter) (Illumina Inc.) compared to the raw clusters was greater than 76% for the whole lane. A total of 2.79 × 10^9^ sequences were obtained for the 30 samples.

### 3.5. Data Analysis

#### 3.5.1. Trimming, Alignment, and Gene Count

FastQ files were uploaded on the Galaxy Europe platform [124] and reads were trimmed with Trimmomatic v0.38.1 [125]. Reads were aligned on the *Phaeodactylum tricornutum* Pt1.8.6 genome (Phaeodactylum_tricornutum.ASM15095v2) with HISAT2 v2.2.1 [126]. Gene count was performed with featureCounts v2.0.1 [127], and 72.1 to 77.1% of the reads were assigned on the reference genome.

#### 3.5.2. Differential Gene Expression Analysis

Differential expression of genes (DEG) analysis (Pt2, Pt3, Pt4, Pt5, Pt6, Pt7, Pt8, Pt9, and Pt10 against Pt1) was conducted with DESeq2 v2.11.40.7 [128]. Visualization of DEGs was made by Volcano Plot with VolcanoPlot v0.0.3 (https://bioconductor.org/packages/devel/bioc/vignettes/EnhancedVolcano/inst/doc/EnhancedVolcano.html, accessed on February 2024) and heatmaps with Clustvis (http://biit.cs.ut.ee/clustvis, accessed on February 2024) [129]. The chosen fold change (FC) was 0.5 < FC > 2 (i.e., −1 < log2FC > 1) with a *p* < 0.05 (i.e., log10*p* > 1.3).

#### 3.5.3. Gene Ontology

Gene ontology was explored using the overrepresentation test performed through ShinyGO 0.77 [130] (pathway database: all available gene set, FDR cutoff 0.05). KEGG annotation was obtained using pathway maps of the KEGG PATHWAY database (https://www.genome.jp/kegg/pathway.html, accessed on February 2024). The genes in all other processes were obtained from UniprotKB and from DiatomicBase (https://www.diatomicsbase.bio.ens.psl.eu/resources, accessed on February 2024) by BLAST after identification in the pathway maps of the KEGG PATHWAY database. Venn diagrams were created using JVenn [131].

### 3.6. Protease Activities Assay

For each accession, 5 mL of cell culture was inoculated at 1 × 10^6^ cells.mL^−1^ in triplicate. On day four after inoculation, cell density was measured as described above and 1 mL of each culture was harvested by centrifugation at 2000× *g* for 15 min at room temperature. Culture supernatants were harvested and cell pellets were resuspended in 500 µL of Tris Buffer 0.1 M pH 7.5. Intracellular proteins were extracted using lysing beads (E-matrix lysing tubes, MP Biomedicals^®^, Fisher Scientific, Illkirch, France) and grounded for 4 cycles of 30 s at 6.5 m·s^−1^ in a FastPrep-24^TM^ homogenizer (MP Biomedicals^®^ Santa Ana, CA, USA). Tubes were centrifugated for 5 min at 10,000× *g*. The supernatant was collected and the previous step was repeated. Protease activities were measured using the “Protease Assay Kit” (Calbiochem^®^ San Diego, CA, USA) according to the manufacturer’s instructions, using 200 µL of culture supernatant and 200 µL of cell extracts.

## 4. Conclusions

Altogether, these results demonstrate that omics approaches are powerful tools to help choose the right chassis and strain for the production of biologics in microalgae. Indeed, in this work, applying RNA-Seq analyses on 10 accessions of *P. tricornutum* allowed us to identify the drawbacks and advantages of each of them for the production of secreted recombinant proteins and glycoproteins, and such approach can contribute to decision-making. Our analysis reveals that the Pt9 accession stands out as the most interesting. In fact, the results show its potential in protein synthesis and export, *N*-glycosylation, as well as in secreted proteases. Other accessions, such as Pt4, were shown to be of interest in terms of *N*-glycosylation, quality control, and proteasomes. Finally, some accessions, such as Pt2 and Pt3, showed no significant differences in any of the pathways analyzed. Concerning hierarchical clustering, in all the pathways explored, Pt2 and Pt3 were always clustered together and this cluster was always more distant from the cluster formed by Pt7 and Pt9. With regard to the genetic elements that enable the expression of recombinant proteins, such as promoters, we showed that in this case, no strain seemed to be universal but that the choice of promoter had to be matched more closely to the accession used, or vice versa. It would be interesting to perform further multi-omics studies, adding proteomics or glycomics, to have a more complete picture and thus a better understanding of the physiology of the *P. tricornutum* accessions from a bioproduction point of view.

## Data Availability

The data for this study have been deposited in the European Nucleotide Archive (ENA) at EMBL-EBI under accession number PRJEB75330 (https://www.ebi.ac.uk/ena/browser/view/PRJEB75330 accessed on 23 July 2024).

## Supplementary Materials

The following supporting information can be downloaded at: https://www.mdpi.com/article/10.3390/md22080353/s1, Figure S1: Generation time of the ten accessions; Figure S2: DEGs analysis in the different accessions of *P. tricornutum*; Table S1: Fold enrichment pathways analysis with DEGs found in Pt2 to Pt10 compared to Pt1. Table S2: Correspondence between proteins names and genes involved in ribosome pathway of *P. tricornutum*. Table S3: Correspondence between proteins names and genes involved in protein export and secretion pathways of *P. tricornutum*. Table S4: Correspondence between enzymes names and genes involved in the protein *N*-glycosylation biosynthesis pathway of *P. tricornutum*. Table S5: Correspondence between proteins names and genes involved in protein quality control pathway of *P. tricornutum*. Table S6: Correspondence between proteins names and genes involved in ribosome pathway of *P. tricornutum*.
