# Supplementary material for: Comparative RNA-Seq of Ten Phaeodactylum tricornutum Accessions: Unravelling Criteria for Robust Strain Selection from a Bioproduction Point of View
Source: Mar Drugs. 2024 Jul 30;22(8):353. doi: 10.3390/md22080353 (PMC11355425; doi:10.3390/md22080353)
Supplement: Supplementary file 1 [file marinedrugs-22-00353-s001.zip › 2024_04_25_Supplemental_Figures_Toustou_et_al.2024_Marine_Drugs_CT_IB.pptx]

## Slide 1
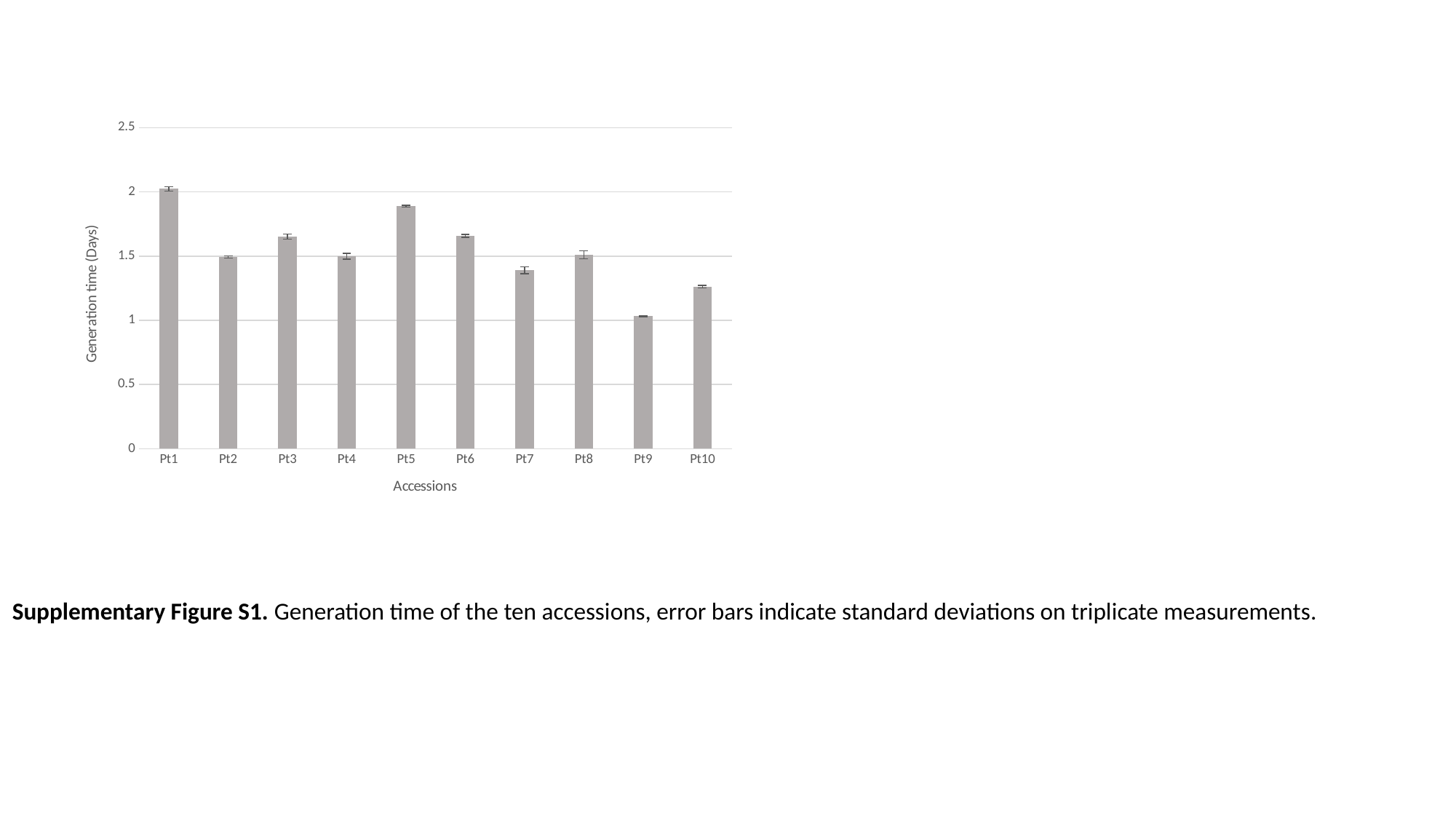

### Chart
| Category | G (Days) |
|---|---|
| Pt1 | 2.0231966741387777 |
| Pt2 | 1.4928864539305304 |
| Pt3 | 1.652317474517152 |
| Pt4 | 1.4986966066160978 |
| Pt5 | 1.8886844156946738 |
| Pt6 | 1.656265664420419 |
| Pt7 | 1.3893509331728708 |
| Pt8 | 1.50946685662009 |
| Pt9 | 1.0323907961869903 |
| Pt10 | 1.2621033877639207 |Supplementary Figure S1. Generation time of the ten accessions, error bars indicate standard deviations on triplicate measurements.

## Slide 2
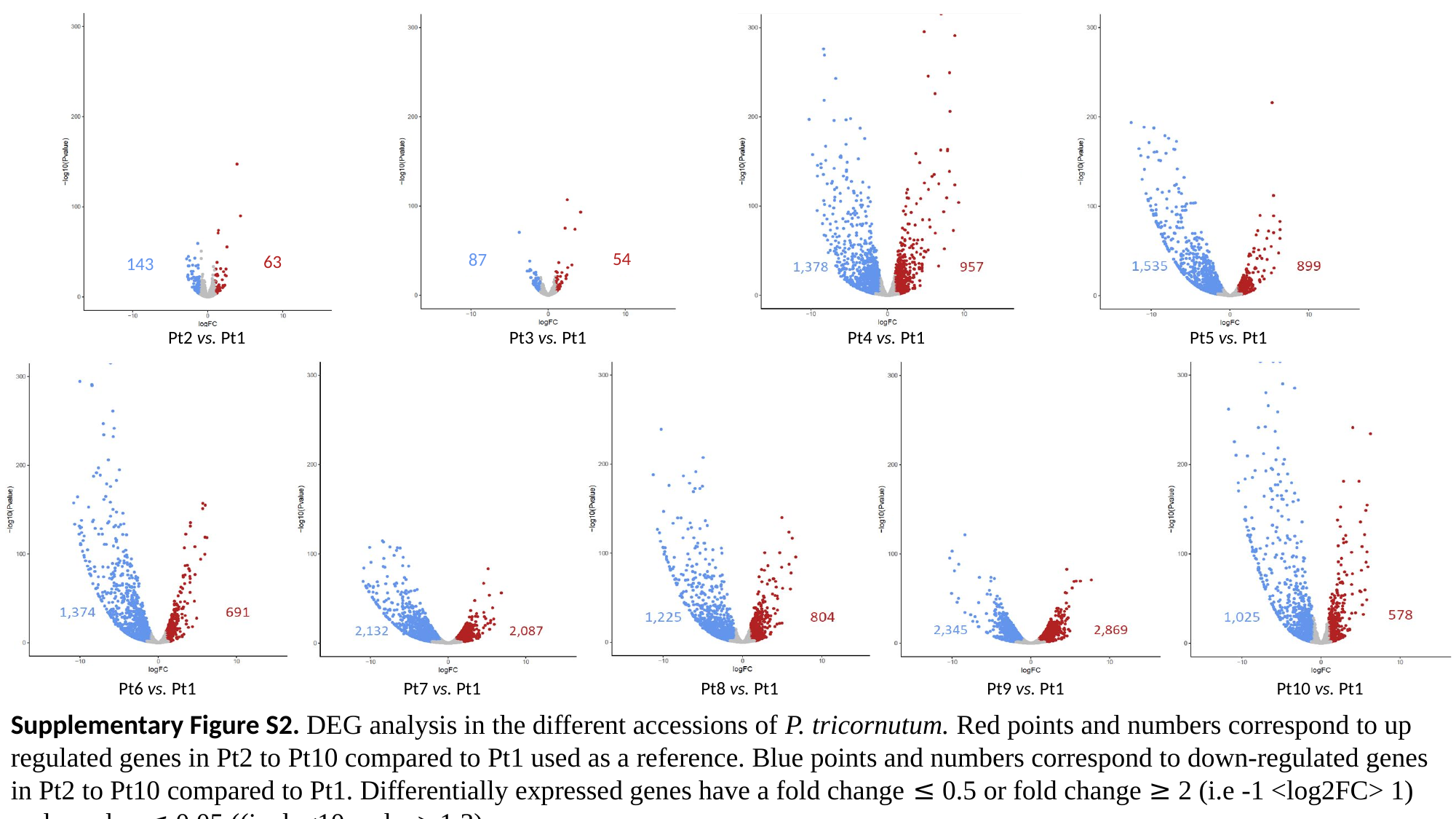

54
87
63
143
Pt2 vs. Pt1
Pt3 vs. Pt1
Pt4 vs. Pt1
Pt5 vs. Pt1
Pt6 vs. Pt1
Pt7 vs. Pt1
Pt8 vs. Pt1
Pt9 vs. Pt1
Pt10 vs. Pt1
Supplementary Figure S2. DEG analysis in the different accessions of P. tricornutum. Red points and numbers correspond to up regulated genes in Pt2 to Pt10 compared to Pt1 used as a reference. Blue points and numbers correspond to down-regulated genes in Pt2 to Pt10 compared to Pt1. Differentially expressed genes have a fold change ≤ 0.5 or fold change ≥ 2 (i.e -1 <log2FC> 1) and p-value ≤ 0.05 ((i.e log10pvalue> 1,3).

## Slide 3
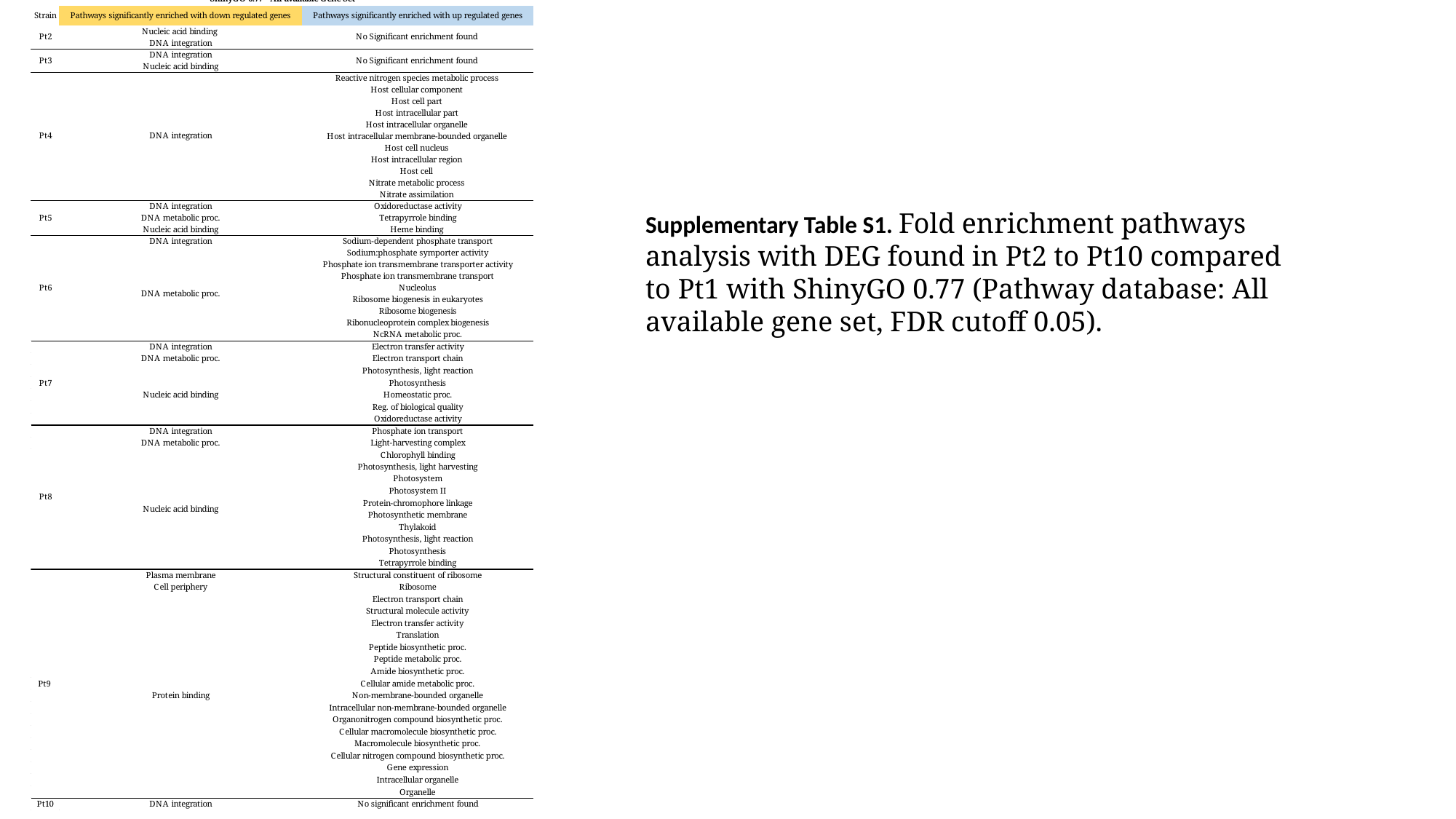

Supplementary Table S1. Fold enrichment pathways analysis with DEG found in Pt2 to Pt10 compared to Pt1 with ShinyGO 0.77 (Pathway database: All available gene set, FDR cutoff 0.05).
